# Jervell and Lange-Nielsen syndrome with novel KCNQ1 and additional gene mutations

**DOI:** 10.1038/s41439-020-00121-x

**Published:** 2020-10-15

**Authors:** Shinichi Matsuda, Yuko Ohnuki, Mayuri Okami, Eriko Ochiai, Shiro Yamada, Kazumi Takahashi, Motoki Osawa, Kenji Okami, Masahiro Iida, Hiroyuki Mochizuki

**Affiliations:** 1 Department of Pediatrics, Tokai University School of Medicine, Isehara, Japan; 2 Department of Medical Ethics, Tokai University School of Medicine, Isehara, Japan; 3 Department of Clinical Genetics, Tokai University Hospital, Isehara, Japan; 4 Department of Otolaryngology, Tokai University School of Medicine, Isehara, Japan; 5 Department of Otolaryngology, Samukawa Hospital, Samukawa, Japan; 6 Department of Forensic Medicine, Tokai University School of Medicine, Isehara, Japan; 7 Department of Pediatrics, Tokai University Oiso Hospital, Oiso, Japan

**Keywords:** Disease genetics, Genetic counselling

## Abstract

We encountered a boy with Jervell and Lange-Nielsen syndrome (JLNS) with compound heterozygous *KCNQ1* mutations, maternal Trp248Phe and a novel paternal mutation, Leu347Arg. His father showed long QT (LQT) and arrhythmia. His mother was asymptomatic with no ECG abnormalities. The proband and his father had an additional mutation (*SNTA1* Thr372Met), which is reportedly related to SIDS. These results suggest that multiple gene mutations influence the phenotype of *KCNQ1* mutation-related arrhythmia.

Jervell and Lange-Nielsen syndrome (JLNS) (OMIM # 220400) is a life-threatening autosomal recessive disorder characterized by a prolonged QT interval on electrocardiography (ECG) and congenital deafness^1,2^. Fatal arrhythmia is triggered by emotional or physical stress. JLNS is caused by homozygous or compound heterozygous mutations in either the *KCNQ1* or *KCNE1* gene. Without clinical intervention, the prognosis of JLNS is poor. JLNS is the most serious of the major variants of long-QT syndrome (LQTS). Nearly 90% of patients are symptomatic, and sudden death occurs in >25% of patients despite beta-blocker therapy. In addition, JLNS patients begin to suffer from cardiac events very early in life. In the first year of life, 15% had already experienced a cardiac event, 50% had experienced a cardiac event by 3 years of age, and 90% had symptoms by 18 years of age^3–5^.

We experienced a case involving a 4-year-old boy with congenital hearing impairment and loss of consciousness. He was born at 36 weeks and 3 days of gestational age by cesarean section delivery due to fetal distress. There were no major complications after birth. A newborn hearing screening test suggested a bilateral hearing disorder, which was confirmed by an otolaryngologist. His developmental milestones were delayed (head control, 8 months; turning over, 10 months; sitting, 12 months), and he was unable to stand at 1 year of age. In addition, he was diagnosed with iron deficiency anemia, and iron administration was started. No cardiac anomalies or arrhythmias were found at this time.

At 1 year and 11 months of age, a genetic test for congenital hearing loss and congenital cytomegalovirus infection was performed, and no abnormalities were suggested. At 2 years of age, cochlear implant surgery was performed. At 4 years and 1 month of age and 4 years and 8 months of age, he had two episodes of sudden poor complexion and loss of consciousness while running for a short time.

At 4 years and 11 months of age, he was running and suddenly fainted and fell onto his back and subsequently experienced convulsions for one minute; then, his consciousness gradually recovered. He was transported to our hospital. When he arrived at the emergency room, his consciousness was clear.

No abnormalities were observed on electroencephalography, head magnetic resonance imaging or magnetic resonance angiography. There was a tendency toward bradycardia for his age, with a heart rate of ~60 beats/min. The prolonged QT interval was diagnosed according to Bazett’s corrected QT interval (QTcB), which was 470–500 ms. A notched T wave was also recognized in three leads on ECG. Echocardiography revealed normal cardiac function with no cardiac malformations. No electrolyte abnormalities were detected.

The patient fulfilled nine points of the long QT (LQT) diagnostic criteria (Schwartz’s score). Furthermore, his symptoms, prolonged QT combined with deafness, QTc 470–500 ms, bradycardia (<60 beats/min), notched T wave in three leads, and T wave alternans, are high-risk factors for cardiac arrest. For the prevention of lethal arrhythmia with prolonged QT, beta-blocker treatment was initiated. Based on the genetic tests described below, he was diagnosed with prolonged QT syndrome due to the *KCNQ1* (LQT1) mutation.

His exercise strength and school lifestyle were restricted according to “the management criteria for children with heart disease” by the Japanese Society of Pediatric Cardiology and Cardiac Surgery. Swimming and related activities were prohibited. In the 3 years after the diagnosis, he had no episodes of syncope.

Because familial LQT syndrome was strongly suspected, we held a multidisciplinary genetic counseling session for the family. Re-examination of the family history revealed that many paternal relatives had prolonged QT intervals. Thus, genetic testing was recommended to his parents (Fig. 1).

**Fig. 1:**
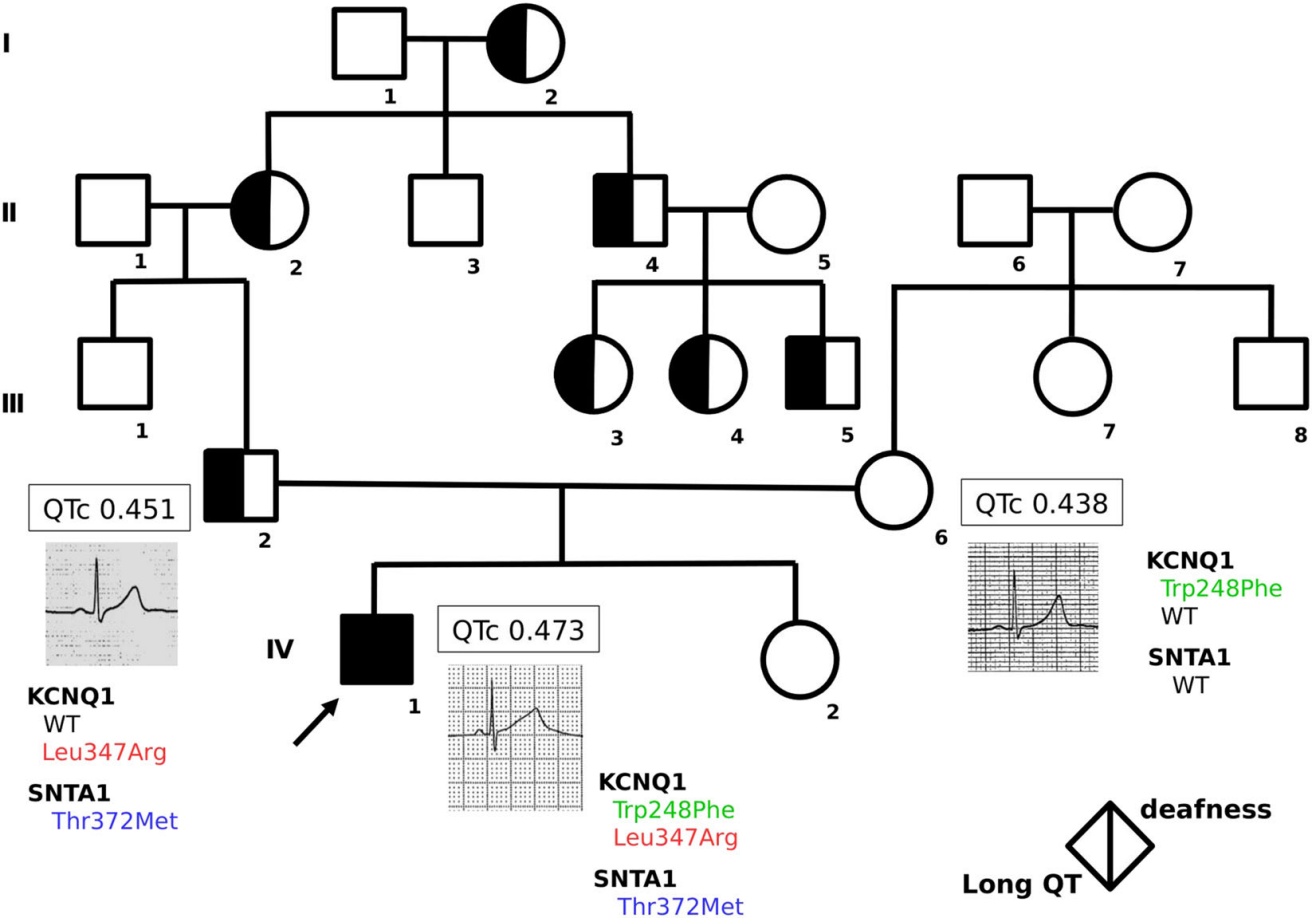
Roman numerals refer to the different generations, and numbers 1–8 identify individuals within each generation. The proband is indicated by the arrow (IV-1). The phenotypes are indicated by half-filled circles or squares; long QT is shown on the left, and deafness is shown on the right. QTc time, electrocardiograms, and the genotypes of the proband and his parents (III-2, III-6) are indicated.

Although many relatives had been diagnosed with QT prolongation, the paternal relatives had no history of syncope or sudden death, and no family history was noted among the maternal relatives.

Genetic testing was carried out for the proband and his parents after obtaining informed consent from the patient’s parents. DNA was extracted using the QIAamp DNA Blood Mini Kit (Qiagen Inc., Hilden, Germany). DNA libraries were constructed using the Ion AmpliSeq^TM^ Library Kit 2.0 (Thermo Fisher Scientific Inc., MA, USA). Libraries were prepared according to the manufacturer’s protocol. Ion AmpliSeq^TM^ Inherited Disease Panel (Thermo Fisher Scientific Inc.) was used as the primer set. Emulsion PCR (emPCR) was performed using the Ion OneTouch 2 instrument (Thermo Fisher Scientific Inc.) with the Ion PGM Template OT2 200 Kit (Thermo Fisher Scientific Inc.) in accordance with the manufacturer’s protocol. Sequencing was performed using the Ion PGM^TM^ Sequencer with the Ion PGM Sequencing 200 Kit v2 (Thermo Fisher Scientific Inc.) and the Ion 316 Chip Kit v2 (Thermo Fisher Scientific Inc.). Sequencing data were analyzed using Torrent Suite Software v 5.0.4 (Thermo Fisher Scientific Inc., MA, USA).

The results showed that the proband (IV-1) had the (NM_000218.3) (c.743_744delGGinsTC: p.Trp248Phe and c.1040T>G: p.Leu347Arg) compound heterozygous mutation in *KCNQ1*. His father (III-2) had the novel mutation p.Leu347Arg in *KCNQ1*. The same amino acid position mutation (c.1040T>C: p.Leu347Pro) was previously reported as a cause of LQTS^6^. His mother (III-6) had the p.Trp248Phe mutation in *KCNQ1*. This variant (rs397508123) has been reported as a cause of LQT by Ohno et al. ^7^. The same amino acid position changes (p.Trp248Arg and p.Trp248Cys) are also reported as causes of LQTS^8,9^. In addition, a mutation in the α1-syntrophin (*SNTA1*) gene (NM_003098.3) c.1115C>T Thr372Met (rs1275683627), which is reported to be related to SIDS^10^, was recognized in the proband and his father.

These three variants are not registered in ClinVar or the 1000 Genomes database. We performed PolyPhen-2 and SIFT analyses, and the results were “probably damaging” and “damaging”, respectively, for all three variants. In the gnomeAD database, the frequency of the variant *SNTA1* c.1115C>T Thr372Met (rs1275683627) was 0.00000 (Asian) and 8.024 × E^−6^ (total population), and two *KCNQ1* variants were not registered. The *KCNQ1* variants were predicted to be likely pathogenic in the guidelines for the interpretation of sequence variants of American College of Medical Genetics^11^ as follows: three moderate (PM2: Absent from controls or at extremely low frequency if recessive, in the 1000 Genomes or gnomeAD databases, PM3: Detected in trans with a pathogenic variant for recessive disorders, PM5: A novel missense amino acid change occurring at the same position as another pathogenic missense change) and two supporting (PP3: multiple lines of computational evidence support a deleterious effect on the gene or gene product, SIFT: damaging, PolyPhen-2: probably damaging, and PP4: patient’s phenotype or family history is highly specific for a disease with a single genetic etiology).

Until now, *KCNQ1*, *KCNH2*, and *SCN5A* have been reported as candidate genes for prolonged QT syndrome that may cause SIDS. *CAV3*, *SCN3B*, *SCN4B*, and *SNTA1* are also candidate genes that may cause SIDS^10,12^. However, as reported by Giudicessi et al. “individuals who harbor the same LQTS-causative mutation often assume vastly different clinical courses in terms of QTc duration and frequency of cardiac events”. LQTSs are not simple Mendelian-inherited disorders^13^.

This was the same in our case. Although the proband’s father had a *KCNQ1* Leu347Arg mutation and showed QT prolongation, he was asymptomatic. All of his relatives who showed QT prolongation were asymptomatic, and in deceased relatives, death was not related to cardiac events. Genetic testing was not conducted for the relatives. On the other hand, although the proband’s mother had the Trp248Phe mutation in *KCNQ1*, which is reported to be a causative mutation of JLNS^7^, there was no apparent QT prolongation, and none of her relatives had QT prolongation. Symptoms only appeared in the proband, who had compound heterozygous mutations Leu347Arg and Trp248Phe in *KCNQ1*.

Interestingly, a mutation in *SNTA1* was also detected in the proband and his father. This gene has been reported to cause SIDS, with an increase in the action potential duration (APD) or QT time, due to an increase in the steady-state component (Late-INa) of the Na+ current in the Na channel of the heart^10^.

The proband and his father had mutations in both *KCNQ1* and *SNTA1* and showed obvious QT prolongation, whereas the father and his relatives had no history of syncope. His mother also had no history of arrhythmia or syncope. Regarding the phenotypic difference between the JLNS child and his parents, Westenskow et al. already reported that “parents who carry 1 loss-of-function mutation of KVLQT1 or KCNE1 generally have few or no symptoms”^14^. In our case, based on the ACMG guidelines, the responsible paternal gene mutation was considered *KCNQ1*. This is supported by the fact that the ECG T wave patterns most closely matched the *KCNQ1* mutation, as previously reported^15,16^. However, the effect of a mutation in *SNTA1* cannot be completely excluded.

According to the above results, it was once again recognized that genetic abnormalities alone cannot predict the prognosis of LQTS. Previous studies on the risk stratification of congenital LQTS^17^ proposed the following high-risk factors: QTc > 500 ms, male sex, and a gene mutation in LQT1, LQT2, and LQT3. In the future, it could be possible to diagnose a patient and select an appropriate treatment according to the combination of gene mutations, not only apparently deteriorative mutations but also slightly to moderately effective mutations, and symptoms confirmed by clinicians. ECG should be repeatedly performed for patients with congenital hearing loss and repeated episodes of syncope to detect LQTS.

We herein report a new combination of genetic mutations that could cause fatal arrhythmia. Our case also suggests that while QT intervals are somewhat inherent, the additional genetic factors that lead to fatal arrhythmia may be heterogeneous.

## Data Availability

The relevant data from this Data Report are hosted at the Human Genome Variation Database at 10.6084/m9.figshare.hgv.2915 10.6084/m9.figshare.hgv.2918 10.6084/m9.figshare.hgv.2921
